# Evaluation of the Peripheral and Central Auditory Systems in Children and Adolescents Before and After COVID-19 Infection

**DOI:** 10.3390/children11121454

**Published:** 2024-11-28

**Authors:** Julia Siqueira, Milaine Dominici Sanfins, Piotr Henryk Skarzynski, Magdalena Beata Skarzynska, Maria Francisca Colella-Santos

**Affiliations:** 1 Faculty of Medical Sciences, State University of Campinas, Campinas 13083-887, SP, Brazil; j171144@dac.unicamp.br (J.S.); mfcolell@unicamp.br (M.F.C.-S.); 2 Department of Speech-Hearing-Language, Universidade Federal de São Paulo, São Paulo 04044-020, SP, Brazil; 3 Post-Graduate Program in Clinical Audiology, Instituto de Ensino e Pesquisa Albert Einstein, São Paulo 05652-000, SP, Brazil; 4 Department of Teleaudiology and Screening, World Hearing Center, Institute of Physiology and Pathology of Hearing, 05-830 Kajetany, Poland; p.skarzynski@csim.pl; 5 ENT Department, Maria Curie-Skłodowska University, 20-031 Lublin, Poland; 6 Center of Hearing and Speech Medincus, 05-830 Kajetany, Poland; m.skarzynska@csim.pl; 7 Department of Otolaryngology, Institute of Sensory Organs, 05-830 Warsaw, Poland; 8 Heart Failure and Cardiac Rehabilitation Department, Medical University of Warsaw, 02-091 Warsaw, Poland; 9 World Hearing Center, 05-830 Kajetany, Poland; 10 Department of Pharmacotherapy and Pharmaceutical Care, Pharmaceutical Department, Medical University of Warsaw, 02-091 Warsaw, Poland; 11 Institute of Sensory Organs, 05-830 Kajetany, Poland

**Keywords:** betacoronavirus, hearing, child, adolescent

## Abstract

COVID-19 is an infectious disease caused by the SARS-CoV-2 virus. During and after COVID-19, audiovestibular symptoms and impairments have been reported. Objectives: This study aimed to investigate the impacts of COVID-19 on the peripheral and central auditory systems of children and adolescents following the acute COVID-19 phase based on behavioral, electroacoustic, and electrophysiological audiological assessments. Methods: This is a primary, prospective, observational, and cross-sectional study of 23 children aged 8 to 15 years who acquired confirmed COVID-19 and who, before infection, had not had any auditory complaints or school complications. The results were compared with pre-pandemic data collected from a similar group of 23 children who had normal peripheral and central hearing and good school performance. Each participant answered a questionnaire about child development, school, and health history and underwent tests including pure-tone audiometry and high-frequency audiometry, imitanciometry, transient evoked otoacoustic emissions, and distortion product otoacoustic emissions. They also received tests of Brainstem Auditory Evoked Potentials, Long Latency Auditory Evoked Potentials, Dichotic Digits Test, Sentence Identification Test, Dichotic Consonant–Vowel Test, Frequency Pattern Test, and Gaps-In-Noise Test. Results: Significant differences were observed between the groups, with the study group showing worse thresholds compared to the control group at both standard audiometric frequencies and at higher frequencies, although both groups were still within normal limits (*p* ≤ 0.05). In addition, the study group had a higher prevalence of absent responses, as identified by otoacoustic emissions and acoustic reflexes. In terms of central auditory performance, the study group showed ABRs with significantly longer latencies of waves I, III, and V compared to the control group. The study group also performed less well on the Dichotic Digits and Pediatric Speech Identification tests. Conclusions: COVID-19 appears to alter the auditory system, both peripherally at the level of the outer hair cells and more centrally.

## 1. Introduction

COVID-19 is an infectious disease caused by the SARS-CoV-2 virus, a novel member of the coronavirus family. The initial cases, originating in the city of Wuhan, China, were reported to the World Health Organization (WHO) in December 2019 [1]. The disease spread globally and was declared a pandemic in March 2020. It persisted until May 2023, when an end to the Public Health Emergency of International Concern relating to COVID-19 was announced [2].

The transmission of COVID-19 primarily occurs through respiratory routes and involves the dispersion of virus-containing droplets. The virus predominantly affects the respiratory system, leading to flu-like symptoms such as cough, fever, and dyspnea. Other vital organs and systems can also be affected, giving rise to complications. The neurological complications experienced by COVID-19 patients suggest the involvement of the peripheral and central nervous systems [3].

After the acute phase of COVID-19 is over, more than a hundred post-COVID-19 symptoms have been reported; many are associated with reduced quality of life and impaired performance of daily activities [4]. Long COVID, or the Post-COVID Condition, is defined by WHO as a condition that occurs in individuals following a SARS-CoV-2 infection, typically around 3 months later, with symptoms lasting for 2 months or more that are not attributable to another diagnosis [5].

Audiovestibular symptoms—hearing loss, tinnitus, and vertigo—are often reported during and after COVID-19 infection. Case studies of adults following COVID-19 infection and treatment have identified sudden hearing loss, either unilateral or bilateral, ranging from mild to severe, often accompanied by tinnitus [6,7,8]. Studies of tissues and ciliated cells associated with the pathogenesis of the virus show evidence that SARS-CoV-2 impairs cochlear function [9,10]. Even asymptomatic adults who have confirmed COVID-19 but have no auditory complaints appear to have damaged hair cells, as evidenced by pure-tone audiometry, tympanometry, and otoacoustic emissions [11].

Turning to the central auditory system, auditory evoked potentials appear to show that COVID-19 affects the brainstem as well. Studies of auditory brainstem responses (ABRs) in adults indicate longer latencies compared to control groups [12,13].

The way COVID-19 manifests, and its effects following the acute phase of the disease, differ between adults and children [14]. For children aged 1 to 18 years, most cases are asymptomatic or exhibit only mild to moderate symptoms. The more common asymptomatic presentation in children may reflect the lower likelihood of underlying conditions such as diabetes, hypertension, or cardiovascular disease [15].

Regarding Long COVID in children and adolescents, studies are scarce, and there is a lack of clarity regarding severity, duration, and incidence. It is evident that more research on this topic is needed [16].

Studies on the impacts of COVID-19 on the auditory system of children have generally been retrospective. Others have assessed neonates born to mothers who were infected with SARS-CoV-2 during pregnancy [17,18,19]. However, one study [20] assessed the peripheral and central auditory systems of 87 participants aged 5 months to 17 years using (depending on their age) otoscopy, pure-tone and speech audiometry, tympanometry, otoacoustic emissions, auditory brainstem responses (ABRs), and Central Auditory Processing tests. No cases of auditory or CAP alterations were found in this group. Nevertheless, the study posited that people who have had the disease should be monitored because there are a variety of symptoms and multiple long-term consequences of COVID-19 [20].

Because of the possibility of long-term effects due to SARS-CoV-2 infection, the aim of this study was to investigate the impacts of COVID-19 on the peripheral and central auditory system of children and adolescents following the acute phase of the disease. Assessments were conducted on average 15 months after infection using behavioral, electroacoustic, and electrophysiological methods.

## 2. Materials and Methods

This is a primary, prospective, observational, and cross-sectional study approved by the Research Ethics Committee of the institution (CAAE: 56309121,7,0000,5404; number 5,454,075). Participants signed the Free and Informed Assent Form, as did their legal guardians.

### 2.1. Participant Selection

The study group (SG) was composed of 23 children and adolescents who had previously suffered from COVID-19. The participants were aged 8 to 15 years, and their previous COVID-19 had been confirmed through laboratory tests (RT-PCR), rapid antigen testing (Ag-RAT), or self-antigen testing (Ag-ST). They had no history of otolaryngological events that could have led to hearing loss; no prior auditory, speech, or language issues; and no academic difficulties before the COVID-19 pandemic.

A retrospective assessment was conducted by the pediatric medical team at a tertiary hospital, identifying patients admitted to the pediatric unit between 2020 and 2023 who had tested positive for COVID-19 during hospitalization. Based on this assessment, electronic medical records were screened based on the study’s inclusion and exclusion criteria. A total of 26 eligible patients were identified. After attempting to contact the guardians, 6 children and adolescents agreed to participate and attended the evaluation. Additionally, recruitment efforts included distributing flyers at health centers and schools, as well as posting invitations on social media. Through these efforts, 21 additional children and adolescents were invited, 17 of whom met the inclusion criteria. This resulted in a total of 23 participants in the study.

The control group (CG) was composed of 23 children and adolescents evaluated prior to the COVID-19 period. The control group included children aged 8 to 15 years with typical development and no otological or auditory complaints. Data for this group were retrieved from the institution’s database and were collected in the same setting and using the same equipment as the study group.

Exclusion criteria for both groups encompassed children and adolescents with developmental disorders, neurological conditions, genetic syndromes, or diseases with otological repercussions, auditory processing disorder, the use of psychoactive medication and ototoxic medication, and a family history of hearing loss.

### 2.2. Procedures

The participants attended the Audiology Laboratories of the institution between November 2020 and November 2023 and were tested in acoustically treated rooms and booths. The legal guardian of each participant answered a questionnaire regarding child development, school performance, and medical history, including COVID-19.

The assessment was conducted an average of 15.4 months after confirmed SARS-CoV-2 infection (SD = 6.8).

Procedures for assessing the peripheral auditory system were as follows:

- Inspection of the external auditory meatus using a Heine otoscope;
- Pure-tone audiometry (thresholds at 0.25, 0.5, 1, 2, 3, 4, 6, and 8 kHz);
- High-frequency audiometry (at 9, 10, 11.2, 12.5, 14, and 16 kHz);
- Tympanometry using a 226 Hz probe tone;
- Acoustic reflex levels, both ipsilateral and contralateral (assessed at 0.5, 1, 2, and 4 kHz);
- Transient otoacoustic emissions (TOAEs) assessed at 1000, 1420, 2000, 2830, and 4000 Hz. Data were collected automatically from 300 stimulus sweeps, with minimum values of 90% stability and 73% reproducibility. To be deemed present, a minimum signal-to-noise ratio (SNR) of 6 dB was required.
- Distortion product otoacoustic emissions (DPOAEs) at 0.5, 1, 1.5, 2, 3, 4, 5, 6, 7, 8, 9, and 10 kHz. The sound stimulus consisted of two pure tones, F1 = 65 dB and F2 = 55 dB, F2/F1 = 1.22, with a minimum SNR of 6 dB and minimum reliability of 98%.

The assessments used an Interacoustics AC40 audiometer, AT235 impedance audiometer, and Titan Otoemission Module (Interacoustics Brasil, São Paulo, Brazil), all with current calibration. Testing protocols and normality criteria followed World Health Organization (2020) guidelines for audiometry, tympanometry, and acoustic reflexes [21].

Behavioral assessment of the central auditory system was conducted using a computer connected to the AC40 audiometer, with tests applied in random order. The tests were administered according to the recommendations of Pereira and Schochat (2011) and Musiek (1994; 2005) [22,23,24] and included the following tests:

- Dichotic Digits Test;
- Sentence Identification Test (PSI or SSI, monotic presentation, SNR −15 dB);
- Dichotic Consonant–Vowel Test (free attention stage);
- Frequency Pattern Test;
- Gaps-In-Noise Test.

Electrophysiological assessments were conducted using the Smart EP module from Intelligent Hearing Systems. Brainstem Auditory Evoked Potentials (Auditory Brainstem Response) focused on waves I, III, and V and were evoked by the presentation of 2000 rarefied polarity click stimuli at an intensity of 80 dB HL, using a band-pass filter of 30–1.5 kHz. Long Latency Auditory Evoked Potentials (LLAEPs) were examined following the protocol proposed by McPherson (1996) and focused on the latency and amplitude of the P300 wave [25]. The rare stimulus was a 2 kHz tone burst with a presentation probability of 20%, and the frequent stimulus was a 1 kHz tone burst, with a presentation probability of 80% and intensity of 70 dB HL. The presentation rate was one stimulus per second for a total of 300 sweeps.

### 2.3. Analysis

In all tests, comparisons were made between the study and control groups, except for the acoustic reflex and TOAE and DPOAE tests, where pre-pandemic data for the control group was missing. In this case, the analysis considered the criteria listed above to classify responses as either present or absent.

Statistical analysis utilized SPSS V26 (2019), Minitab 21.2 (2022), and Excel Office 2010. The significance level was set at 5% (*p* ≤ 0.05), highlighted in bold in the tables. The normality of the quantitative outcome variables was tested using the Kolmogorov–Smirnov test, which revealed that the data did not follow a normal distribution. To characterize the distribution of sex, the Z-test for Two Proportions was used. Comparisons between the study and control groups were made using the Mann–Whitney U-test. Because there was no statistically significant difference between the ears, the data for the peripheral and central auditory evaluation tests was grouped and analyzed jointly (*N* = 46), except for otoacoustic emissions and the Dichotic Consonant–Vowel Test.

## 3. Results

The study group comprised 23 participants aged 8 to 15 years (mean = 11.4, SD = 2.0), with 15 (65%) being male, showing a statistically significant difference in gender distribution (*p* = **0.039**).

The symptoms reported by the participants during and after the acute phase of COVID-19 are presented in Table 1. The reported post-COVID-19 symptoms persisted until the time of evaluation.

In this phase, 13 (56.5%) participants reported using one or more medications, such as analgesics (*N* = 12, 52.2%), antipyretics (*N* = 12, 52.2%), antibiotics (*N* = 4, 17.4%), anti-inflammatories (*N* = 3, 13.0%), and antiparasitics (*N* = 1, 4.3%). There were two participants (8.7%) who reported they needed hospitalization during the acute phase.

### 3.1. Peripheral Behavioral Auditory Assessment

In pure-tone audiometry at frequencies from 0.25 to 8 kHz, all participants (*N* = 23, 100%) had auditory thresholds ≤ 20 dB HL. Regarding high-frequency audiometry, all subjects (*N* = 20, 100%) showed thresholds ≤ 25 dB HL at all tested frequencies, from 9 to 16 kHz. Table 2 lists the thresholds at frequencies from 0.25 to 16 kHz obtained in the control and study groups.

In tympanometry, type A curves were identified in 45 ears (97.8%) and a type Ar (middle ear volume below 0.3 mL) in 1 ear (2.2%).

**Figure 1:**
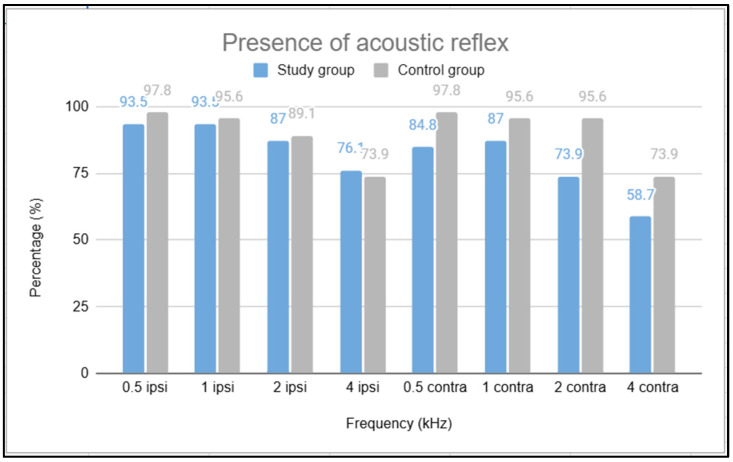
Presence of the acoustic reflex by sex and age in the study group and the control group (each with *N* = 23).

For the study of acoustic reflexes, Table 3 presents the results of ipsilateral reflexes by frequency for the study group (SG), classified as either present or absent. It also shows contralateral reflexes classified as present at normal levels, present at increased levels, or absent, according to the classification by Jerger et al. (1972) [26]. Figure 1 illustrates the percentages of ipsilateral and contralateral reflexes by frequency for the SG and CG.

### 3.2. Peripheral Electroacoustic Auditory Assessment

Table 4 presents the present or absent results obtained in the TOAE test for each investigated frequency (18 participants), and Table 5 shows results for DPOAEs (20 participants).

### 3.3. Central Behavioral Auditory Assessment

The results of the control and study groups for the Dichotic Digits Test (DDT), Pediatric Sentence Identification Test (PSI/SSI), Frequency Pattern Test (FPT), Gaps-In-Noise Test (GIN), and Dichotic Consonant–Vowel Test (DCVT) are presented in Table 6.

### 3.4. Central Electrophysiological Auditory Assessment

Table 7 displays the latencies (ms) of waves I, III, and V of the ABRs, as well as the latency (ms) and amplitude (µV) values of the P300 wave obtained in the control and study groups.

## 4. Discussion

The present study aimed to assess the impacts of COVID-19 on the peripheral and central auditory systems of 23 children and adolescents. Of the sample, 15 were males. Studies indicate that males are more susceptible to COVID-19 than females, even in children, probably due to hormonal and chromosomal factors [27].

Studies on the prevalence of auditory and vestibular symptoms in children after SARS-CoV-2 infection have reported the presence of ear fullness, earache [19,28], and tinnitus [29]. These manifestations were also reported by our participants. The most commonly reported post-COVID symptom here was dizziness, which has also been frequently reported by patients with COVID-19 [30,31,32,33].

Viruses such as SARS-CoV-2 can damage the nervous system due to their affinity for nerve cells, resulting in neurological symptoms such as dizziness and vertigo. The audiovestibular symptoms may be explained by the virus’s infiltration into the central nervous system via ACE2 receptors, the intense systemic cytokine immune response triggered by the virus, and brainstem dysfunction resulting from neuroinflammation [34,35]. Other factors may have also contributed to dizziness, such as the social and emotional repercussions of the COVID-19 pandemic and the use of ototoxic medications [36,37].

Medications and ototoxic substances can damage hair cells and the vestibular system, manifesting initially through changes in high-frequency hearing and later affecting medium and low frequencies. This is because ototoxicity primarily affects the basal portion of the cochlea [38]. To check whether our results could have been affected by ototoxic medications, the medications reported by participants to have been used during COVID-19 were examined. Only one was identified as ototoxic: azithromycin, which was used by three participants. However, azithromycin ototoxicity is rare, occurring only after prolonged use at high doses, such as in treatment for infections related to acquired immunodeficiency syndrome, which was not the case for these three participants.

All participants presented auditory thresholds within the normal range bilaterally, according to the WHO classification [21]. However, when the two groups were compared, there was a statistically significant difference at most frequencies, with higher values in the COVID-19 group. Similarly to our results, another study conducted on adults showed normal thresholds in the COVID-19 group but a statistically significant difference between groups for standard and high-frequency thresholds [13]. For children, however, we have found only one relevant study but it did not compare measured auditory thresholds with a control group [20].

The highest number of absent acoustic reflex responses in the study group occurred at 2000 and 4000 Hz, and in the comparison between the SG and CG, fewer present responses were obtained at all frequencies tested for the contralateral reflex, pointing to changes in the reflex pathway resulting from SARS-CoV-2 infection.

There was a notable higher absence of TOAEs and DPOAEs at high frequencies. In the case of DPOAE, a greater absence of emissions was also observed at 500 and 1000 Hz. However, this absence may be explained by noisier measurements at low frequencies [39].

Studies on the SARS-CoV-2 virus reveal that the epithelium of the middle and inner ear has receptors for angiotensin-converting enzyme 2 and transmembrane serine protease 2, making entry of the SARS-CoV-2 virus into hair cells possible [9]. Innervation of the cochlear base is composed of high-frequency fibers, so viral damage to the hair cells at the base may explain the absence of otoacoustic emissions found in this study. This may also explain alterations in otoacoustic emissions described by other studies of adults [10,11,12].

Beyond damaging the peripheral system, SARS-CoV-2 infection may also affect the central nervous system [40] by entering through angiotensin-converting enzyme 2 and spreading to the brainstem through peripheral nerves [41]. Another possibility is that SARS-CoV-2 alters tissues in the brainstem, leading to inflammation and neurodegeneration [42].

Our behavioral assessment showed statistically lower performance for the COVID-19 group in the Dichotic Digit Test and the Pediatric Sentence Identification Test, indicating a possible impairment in central auditory processing. The other behavioral tests—Frequency Pattern Test, Gaps-In-Noise Test, and Dichotic Consonant–Vowel Test—did not show any statistically significant differences. Among the symptoms resulting from central auditory processing deficits are difficulties in auditory comprehension, following instructions, and maintaining attention [43]. In the present study, these symptoms were reported by participants after the acute phase of COVID-19 and may explain the study group’s deficits in two of the tests. It therefore appears crucial, post-COVID, to closely monitor children’s complaints related to attention and comprehension, as these issues can impact development and learning. If a child has such symptoms, a central auditory processing (CAP) assessment is recommended so that rehabilitation strategies, such as speech therapy and acoustically controlled training, can be implemented.

Regarding the ABRs, significantly increased latencies were observed in the study group compared to the control group for waves I, III, and V, which may indicate alterations in the auditory pathway from the auditory nerve to the brainstem. Two studies conducted among adults have also shown increased latencies in the experimental group [12,13]. However, two other studies conducted with children up to 17 years of age have reported ABR results within normal values [20,44].

In the present study, there was no statistically significant difference between the study group and the control group for the values of the P300, both in terms of latency and amplitude.

### Limitations

The acoustic reflex and otoacoustic emission results were not compared with the control group due to the lack of pre-pandemic data for this group, which constitutes a limitation of the study.

Another limitation was the absence of an assessment of vestibular function. Including this aspect may provide a broader understanding of the auditory findings.

Changes in high-frequency auditory thresholds and otoacoustic emissions may also be attributed to constant noise exposure. However, noise exposure was not controlled or investigated in the participants of the control and study groups, representing a limitation of this study.

The limited sample size also represents a limitation of this study, potentially impacting the representativeness of the results. Expanding the sample in future studies will help to validate these results and improve their applicability.

The research group will continue the study by identifying children who had COVID-19, increasing the sample size, and conducting a comprehensive evaluation of the peripheral and central auditory systems to confirm the findings and strengthen the results.

## 5. Conclusions

This study established hearing limits within normal standards for all participants in the study group but with significant differences compared to the control group.

Although they do not represent hearing loss, absent responses were identified at high frequencies in otoacoustic emissions and in the acoustic reflex research, which indicates changes in hair cells and afferent and efferent pathways, suggestive of having been caused by COVID-19.

As with the evaluation of the peripheral system, some tests of the central auditory system evaluation showed results with significant differences compared to the control group.

Several alterations in the auditory system of the study group were observed, and it is suggested that these are results of COVID-19 infection. The measured impairments ranged from peripheral ones affecting the outer hair cells of the cochlea to effects on more central pathways.

In light of our results, any anamnesis should inquire into SARS-CoV-2 infection and whether there were audiovestibular symptoms during or after the acute phase of the disease. If so, peripheral and central audiological assessments should be recommended to determine whether there has been any permanent damage to the auditory system.

## Figures and Tables

**Table 1:** Symptoms of the study group (*N* = 23) during the acute phase of COVID-19 (middle column) and after the acute phase (right column).

Symptoms	During Acute Phase *N*	After Acute Phase *N*
Fever	14	0
Rhinorrhea	9	0
Headache	6	0
Cough	6	0
Body aches	5	0
Sore throat	4	0
Shortness of breath	2	0
Fatigue/weakness	2	0
Diarrhea	1	0
Dizziness	1	4
Vomiting	1	0
Loss of appetite	1	0
Decreased taste	1	0
Tinnitus	0	3
Memory difficulty	0	3
Hearing difficulty	0	2
Inattention	0	2
Comprehension difficulty	0	1
Ear fullness	0	1
No symptoms	3	9

*N*: Number of participants.

**Table 2:** Thresholds of pure-tone audiometry and high-frequency audiometry in both the control group and the study group.

		Mean	Median	SD	Q1	Q3	IQR	*N*	CI	*p*-Value
250 Hz	Control	7.28	5	5.02	5	10	5	46	1.45	0.172
	Study	9.46	10	7.17	5	15	10	46	2.07	
500 Hz	Control	5.65	5	4.79	5	10	5	46	1.38	**0.028**
	Study	8.04	10	5.00	5	10	5	46	1.44	
1000 Hz	Control	4.35	5	4.67	5	5	0	46	1.35	**0.033**
	Study	6.41	5	4.17	5	10	5	46	1.21	
2000 Hz	Control	3.91	5	5.57	0	10	10	46	1.61	0.132
	Study	5.87	5	4.38	5	10	5	46	1.27	
3000 Hz	Control	4.46	5	5.80	0	10	10	46	1.67	**0.022**
	Study	7.28	5	4.56	5	10	5	46	1.32	
4000 Hz	Control	4.35	5	4.03	5	5	0	46	1.16	**0.019**
	Study	6.63	5	4.35	5	10	5	46	1.26	
6000 Hz	Control	3.59	5	4.55	0	5	5	46	1.32	0.359
	Study	4.67	5	7.99	0	10	10	46	2.31	
8000 Hz	Control	4.24	5	4.94	0	5	5	46	1.43	**0.043**
	Study	6.52	5	6.57	0	10	10	46	1.90	
9000 Hz	Control	1.85	0	5.31	0	5	5	46	1.53	**0.003**
	Study	5.50	5	6.39	0	10	10	40	1.98	
10,000 Hz	Control	0.98	0	5.12	−5	0	5	46	1.48	0.497
	Study	1.13	0	10.09	−5	5	10	40	3.13	
11,200 Hz	Control	0.65	0	6.20	−3.75	5	8.75	46	1.79	**0.012**
	Study	5.75	5	9.44	−1.25	11.25	12.5	40	2.93	
12,500 Hz	Control	0.00	0	6.67	−5	5	10	46	1.93	**0.005**
	Study	4.88	5	7.72	10	10	10	40	2.39	
14,000 Hz	Control	−4.24	−5	6.50	−10	0	10	46	1.88	**<0.001**
	Study	2.00	0	7.91	−5	5	10	40	2.45	
16,000 Hz	Control	−12.50	−15	7.73	−20	−5	15	46	2.23	**0.006**
	Study	−6.88	−5	10.04	−15	−3.75	11.25	40	3.11	

SD: standard deviation; Q1: first quartile; Q3: third quartile; IQR: interquartile range; *N*: number of ears; IC: confidence interval. Bold: values with statistically significant differences.

**Table 3:** Responses present and absent in ipsilateral and contralateral reflexes by frequency in SG.

Frequency (Hz)		500	1000	2000	4000
Present ipsilateral reflexes	*N*	43	43	40	35
	%	93.5	93.5	87.0	76.1
Absent ipsilateral reflexes	*N*	3	3	6	11
	%	6.5	6.5	13.0	23.9
Contralateral reflexes present at normal levels	*N*	38	36	32	21
	%	82.6	78.3	69.6	45.6
Contralateral reflexes present and elevated	*N*	1	4	2	6
	%	2.2	8.7	4.3	13.1
Absent contralateral reflexes	*N*	7	6	12	19
	%	15.2	13.0	26.1	41.3

Hz: Hertz; *N*: number of participants; %: percentage value.

**Table 4:** Presence and absence of TOAE by frequency in the study group.

Frequency (Hz)		1000	1420	2000	2830	4000
Right ear present	*N*	18	18	17	16	15
	%	100	100	94.4	88.9	83.3
Right ear absent	*N*	0	0	1	2	3
	%	0	0	5.6	11.1	16.7
Left ear present	*N*	17	18	16	13	14
	%	94.4	100	88.9	73.2	77.8
Left ear absent	*N*	1	0	2	5	4
	%	5.6	0	11.1	27.8	22.2

*N*: number of participants; %: percentage.

**Table 5:** Presence and absence of DPOAE by frequency in the study group.

Frequency (Hz)		500	1000	1500	2000	3000	4000	5000	6000	7000	8000	9000	10,000
Right ear present	*N*	5	13	20	18	18	20	20	20	18	14	13	14
	%	25	65	100	90	90	100	100	100	90	70	65	70
Right ear absent	*N*	15	7	0	2	2	0	0	0	2	6	7	6
	%	75	35	0	10	10	0	0	0	10	30	35	30
Left ear present	*N*	4	16	20	19	20	19	19	19	19	16	16	17
	%	20	80	100	95	100	95	95	95	95	80	80	85
Left ear absent	*N*	16	4	0	1	0	1	1	1	1	4	4	3
	%	80	20	0	5	0	5	5	5	5	20	20	15

*N*: number of participants; %: percentage.

**Table 6:** Results of central auditory assessment tests in the study group and the control group.

		Mean	Median	SD	Q1	Q3	IQR	*N*	CI	*p*-Value
DDT (%)	Control	97.77	100	3.54	97.5	100	2.5	46	1.02	**0.047**
	Study	96.82	97.5	3.38	95	100	5	46	0.98	
PSI/SSI (%)	Control	70.65	70	10.63	60	80	20	46	3.07	**0.046**
	Study	64.35	60	16.69	60	70	10	46	4.82	
FPT (%)	Control	74.00	80	22.75	60	92.25	32.25	46	6.57	0.759
	Study	74.88	80	22.90	60	98.25	38.25	42	6.93	
GIN (ms)	Control	4.89	5	0.60	5	5	0	46	0.17	0.716
	Study	5.50	5	2.55	4	6	2	34	0.86	
DCVT Right (%)	Control	12.52	12	2.89	10	15	5	23	1.18	0.237
	Study	11.22	11	3.13	8	13.5	5.5	23	1.28	
DCVT Left (%)	Control	6.22	6	3.10	4	8	4	23	1.27	0.314
	Study	7.33	7	2.55	5	9	4	23	1.04	

%: percentage; ms: milliseconds; SD: standard deviation; Q1: first quartile; Q3: third quartile; IQR: interquartile range; *N*: number of ears; CI: confidence interval. Bold: values with statistically significant differences.

**Table 7:** Results of the values of waves I, III, and V as well as latency and amplitude of the P300 wave in the control and study groups.

		Mean	Median	SD	Q1	Q3	IQR	*N*	CI	*p*-Value
ABRs-I (ms)	Control	1.64	1.63	0.10	1.58	1.70	0.12	46	0.03	**<0.001**
	Study	1.86	1.83	0.27	1.75	1.90	0.15	46	0.08	
ABRs-III (ms)	Control	3.82	3.84	0.11	3.75	3.91	0.16	46	0.03	**<0.001**
	Study	4.09	4.08	0.26	3.92	4.15	0.23	46	0.07	
ABRs-V (ms)	Control	5.54	5.58	0.20	5.44	5.68	0.24	46	0.06	**<0.001**
	Study	5.92	5.88	0.28	5.75	6.07	0.32	46	0.08	
P300 (ms)	Control	312.8	321	54.4	266.5	353.5	87	46	15.7	0.437
	Study	300.9	293.5	38.4	275.3	326.5	51.3	32	13.3	
P300 (µV)	Control	7.66	6.82	3.69	4.41	9.64	5.23	46	1.07	0.268
	Study	7.05	5.99	4.59	3.39	10.51	7.12	32	1.59	

ms: milliseconds; µV: microvolt; Q1: first quartile; Q3: third quartile; IQR: interquartile range; *N*: number of ears; CI: confidence interval. Bold: values with statistically significant differences.

## Data Availability

The original contributions presented in the study are included in the article, further inquiries can be directed to the corresponding author.
